# Not All Injuries Are the Same: Different Patterns in Sports Injuries and Their Psychosocial Correlates

**DOI:** 10.3390/sports11120237

**Published:** 2023-12-01

**Authors:** Tabea Werner, Alena Michel-Kröhler, Stefan Berti, Michèle Wessa

**Affiliations:** 1 Department of Clinical Psychology and Neuropsychology, Institute of Psychology, Johannes Gutenberg-University Mainz, 55122 Mainz, Germany; wtabea@uni-mainz.de (T.W.); kroehler@uni-mainz.de (A.M.-K.); berti@uni-mainz.de (S.B.); 2 Research Group Wessa, Leibniz-Institute for Resilience Research, 55122 Mainz, Germany

**Keywords:** cluster analysis, model of stress and athletic injury, history of stressors, personality, coping resources, sense of coherence, self-compassion, stress

## Abstract

Sports injuries are ubiquitous and can have far-reaching consequences for athletes (e.g., health, performance). Previous studies have examined various psychosocial influencing factors (e.g., stress), but have mostly focused on only one or two injury characteristics (e.g., frequency), neglecting the broader injury pattern. Thus, the present study aimed to obtain a more differentiated picture of potentially different injury patterns and related profiles of psychosocial factors. We investigated a sample of 213 athletes from a cross-sectional online study. Current injury status, frequency, severity, chronicity, medical treatment, and rehabilitation measures were subjected to cluster analysis indicating a 3-cluster solution with predominantly chronically injured athletes (n = 54), athletes not seeking treatment (n = 62), and athletes utilizing medical treatment and rehabilitation (n = 97). Building on the Model of Stress and Athletic Injury, we subsequently conducted three multivariate analyses of variance (MANOVAs) to examine whether the obtained clusters differed in terms of personality factors (e.g., athletic identity), history of stressors (e.g., life events), and coping resources (e.g., self-compassion). We observed significant differences in all three categories of psychosocial variables implying different intervention possibilities for different injury patterns in the future.

## 1. Introduction

Every minute, on average, four people get injured during sports activities in Germany, summing up to around 2,000,000 sports injuries annually in Germany [1]. The number of injuries in Europe is even higher with approximately 4.5 million annual hospital treatments of sports-injured athletes aged 15 and older [2] and 2.6 million people being treated medically in an outpatient setting [2]. These figures illustrate that sports injuries are an almost inevitable part of regular sports participation [3] and that the effective management of sports injuries has a high potential to improve athletic practices. A greater knowledge of the factors influencing the development or maintenance of sports injuries can, for example, lead to the development of effective therapeutic and preventive strategies. However, little attention is often paid to the complexity of injury patterns in sports, as the focus is mostly on the medical perspective. Therefore, our aim here is to provide a more detailed characterization of sports injuries and their associated psychosocial factors.

According to the International Olympic Committee (IOC) Injury and Illness Epidemiology Consensus Group and associated researchers, an injury is “tissue damage or other derangement of normal physical function due to participation in sports, resulting from rapid or repetitive transfer of kinetic energy“ [4] (p. 27). Hence, an injury is either caused by a sudden trauma damaging ligaments, muscles, and so forth, or caused by overuse, practicing the same movements repeatedly, and for example, leading to micro traumata. These micro traumata may not be damaging at first sight but usually, combined with too little recovery, sum up over time leading to larger damage [5]. 

Sports injuries may have multiple and far-reaching consequences for individuals, clubs, and associations, as well as society. First, sports injuries can lead to decreased psychological and physiological well-being [6] as they may impact quality of life and lead to serious and long-term effects on physical [7] as well as mental health (e.g., anxiety, depression) [6,8]. Second, sports injuries often influence performance, usually leading to performance deterioration and fewer competitions won by teams having more injuries [9]. Third, injuries may lead to negative financial consequences for the individual (e.g., no extended contract) but also for sports teams and organizations (e.g., being less successful, gaining less prices) [10,11] with, for example, costs in the range of GBP 45 million per season in the English Premier League [10] to 610 million euros for the clubs of the top five European leagues [12]. Fourth, sports injuries can be career-changing events, leading to missing once-in-a-lifetime opportunities (e.g., Olympic Games) or to career termination. With this, sports injuries are the leading cause of retirement for top-level athletes [13]. 

With these consequences in mind, previous studies have investigated causes and predictors of sports injuries to develop appropriate prevention and rehabilitation measures. However, previous studies have mostly focused on only one characteristic of injuries when predicting sports injuries or success of rehabilitation. They usually used individual criteria of a person’s injury history, either focusing on a specific diagnosis (e.g., anterior cruciate ligament (ACL) injury) [14] or considering the frequency of injuries, injuries per exposure hours, or injury-related time loss [15,16,17], and examined predictors of these characteristics. Focusing only on one injury characteristic goes along with several limitations [18]. For example, Bahr et al. [18] argue that incidence and severity should be combined to a measure of injury burden to derive a better interpretation of injury data and consequently develop and implement appropriate measures. Additionally, overuse injuries do not tend to be associated with time loss due to injury at all [19]. In general, focusing on one characteristic ignores the fact that injuries are embedded in a broader context with, for example, varying cause, frequency of occurrence, severity, treatment measures, consequences, or recovery time. Therefore, as a first step, the present study aims to characterize sports injuries in more detail, assessing different injury characteristics (e.g., frequency, severity, treatment), and examining them in an exploratorily manner to determine whether participants can be clustered into different injury patterns. 

**Figure 1:**
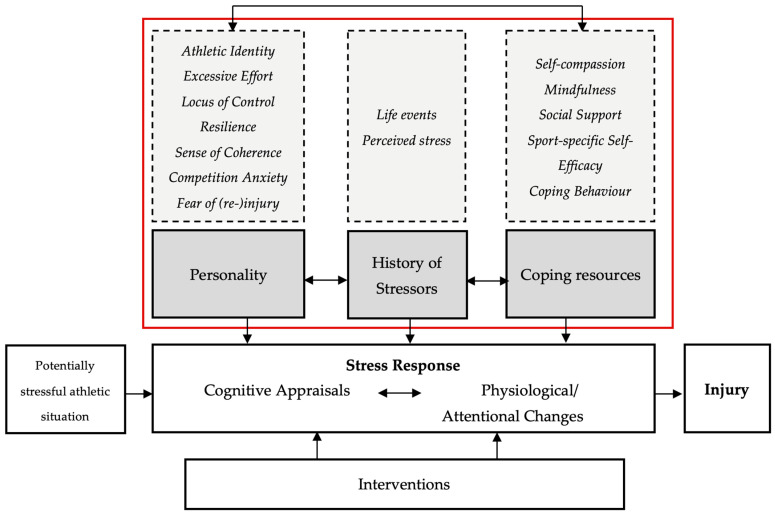
Adapted version of the Model of Stress and Athletic Injury by Williams and Andersen [21]. The model highlights the importance of psychosocial factors in the context of sports injuries.

If there are different patterns of injuries, the question arises whether these patterns can be predicted so that, for example, appropriate prevention measures can be implemented. Previous research has mostly focused on physical (e.g., weather, surface, equipment) and biological (e.g., nutrition, recovery status, training load) causes and predictors of sports injuries and neglected psychosocial factors so that they are underrepresented in research and applied practice [20]. However, an increasing number of studies have elucidated the important role of psychosocial factors beyond physical and biological ones in the sports injury process, spanning from the risk of injury over the response and rehabilitation to return to sport or retirement [8,15,20]. Thus, another aim of this study was to investigate whether individuals with a respective injury pattern systematically differ on psychosocial variables. According to Wiese-Bjornstal [8] “sport psychology is defined as the cognitions, affects, and behaviors of sport participants, and sport socioculture as the social and cultural structures, climates and processes influencing sport participants.” (p. 103) [8]. Thus, psychosocial variables range from personality traits, attitudes, and (coping) behaviors to stress experiences and norms and rules in the cultural context of each sport. To name just one of many conceivable examples representing psychosocial variables in the context of sports injuries, a person who is frequently injured and experiences a high burden may go through more fear of reinjury than a person who is less frequently injured or experiences injuries that can be treated well. Examining psychosocial factors can therefore help to: (1) predict injury occurrence and dealing; (2) derive appropriate interventions; and (3) ultimately improve athletes’ health, performance, and career. In selecting relevant psychosocial factors, we predominantly relied on one of the most frequently cited models in psychological sports injury research that focuses on the predictors of traumatic injuries: the Model of Stress and Athletic Injury (see also Figure 1) [21]. The model was developed as a framework to describe the occurrence of traumatic injuries, therefore including only injuries associated with a known trauma (e.g., bending over in a hole) and not injuries caused by overuse. The model assumes that there are potential stressful situations for athletes leading to specific cognitive appraisals and a related stress response. These appraisals and the stress response are influenced by the personality, history of stressors, and coping resources of athletes. The final stress response influences the likelihood of becoming injured and can be influenced by interventions. 

The current study only uses the categories personality, history of stressors, and coping resources (grey background in Figure 1) as a foundation for analyses of differences between injury patterns. Chosen psychosocial variables of the respective pillars are presented in the dashed boxes and further described in Table 1. 

Many studies have focused on the intermediate pillar of the model relating to stress symptoms [22] and history of stressors as risk factors for an increasing likelihood of sports injuries [15] with daily hassles [23], major life events [24], particularly negative life events [25], and high life stress in general [26] as influencing factors. In a meta-analysis from Ivarsson et al. [15], stress response and history of stressors had the highest correlation with sports injury rates.

**Table 1:** Definition of selected psychosocial variables as defined by the Model of Stress and Athletic Injury [21] and their relation to sports injuries.

Psychosocial Variable	Definition	Possible Relation to Sports Injuries
**Personality**		
Athletic identity	“the degree to which an individual identifies with the athlete role” [27] (p. 1)	High athletic identity can lead to pressure to fulfill this role even though one does not feel well. It can also lead to discrepancies between self-perception and reality when being injured.
Excessive effort	People going beyond their limits to achieve a higher goal [28] (translated by authors)	High excessive effort may elevate the risk of becoming injured due to regularly ignoring boundaries.
Locus of control	“a personal belief about whether outcomes of behavior are determined by one’s actions or by forces outside one’s control.” [29] (p. 7)	Can either increase or decrease level of stress and may especially be important in the attribution of causality of injury.
Resilience	“the ability to “bounce back” from stress” [30] (p. 2)	As stress is a major risk factor for sports injuries, resilience can help to decrease injury risk and help athletes to cope better with injuries.
Sense of coherence	“a belief that the world is meaningful, manageable, and comprehensible” [31] (p. 612)	High levels possibly decrease stress levels and are beneficial in rehabilitation.
Competition anxiety	“a tendency to perceive competitive situations as threatening and to respond to these situations with A-state” [32] (p. 11)	May elevate the risk of becoming injured by elevated stress levels and with that, increased muscle tension or disrupted attention.
Fear of (re-)injury	“an excessive, irrational, and debilitating fear of physical movement and activity resulting from a feeling of vulnerability to painful injury or reinjury.” [33] (p. 36)	May elevate the risk of becoming injured by elevated stress levels and with that, increased muscle tension or disrupted attention, and limits rehabilitation progress.
**History of stressors**		
Life events	“social events requiring change in ongoing life adjustment” [34] (p. 213)	Possibly elevating the stress level and thus increasing the risk of injury.
Stress	There are “two major components of stress: a) stressors in terms of environmental conditions, and b) the person’s reaction to stress” [35] (p. 78)	Increasing the risk of injury by increased muscle tension or disrupted attention.
**Coping resources**		
Self-compassion	“a positive stance toward oneself when things go badly” [36] (p. 115)	Possible protective factor and facilitates dealing with injuries or setbacks in rehabilitation.
Mindfulness	“the tendency to be attentive to and aware of present-moment experiences in daily life” [37] (p. 59)	Possible protective factor by decreasing stress levels and assisting in recognizing/ acknowledging one’s own boundaries.
Social support	“available others to whom one can turn in times of need”[38] (p. 5)	Especially important during rehabilitation by facilitating dealing with injuries or setbacks in rehabilitation.
Sport-specific self- efficacy	“belief that one is capable of sticking to an exercise program, even under unfavorable circumstances” [39] (p. 141)	Eventually beneficial in rehabilitation by increasing adherence; combined with a high excessive effort also a potential risk factor.
Coping	“behaviours […] to alleviate the stressful impact of a situation, either by altering characteristics of the situation or by regulating their emotional reactions to it” [40] (p. 229)	High coping skills can help to decrease injury risk by decreasing stress levels and help athletes to cope better with injuries.

In addition to the stress response and history of stressors, several coping resources (third pillar of the Model of Stress and Athletic Injury) have shown to be associated with a decreased risk of injury. For example, in a study with football players coping with adversity (as defined by the Athletic Coping Skills Inventory–28 [41]), it explained more variance of injury occurrence and days lost due to injury than history of previous injury [42]. Besides coping behavior, social support has been proven to be a coping resource, especially during rehabilitation [43,44] but also as a stress-buffering factor [45,46]. Further, coping resources such as mindfulness, self-compassion, and self-efficacy received more attention in the past years as their influence on stress and health has been shown in the general and working population [47,48,49,50]. In the context of sports injuries, several mindfulness(-based) interventions significantly decreased the risk of becoming injured [51,52]. Although Huysman and Clement [53] did not find a significant relationship between self-compassion and injury reduction, self-compassion might be helpful in dealing with sports injuries and setbacks in the rehabilitation process [54]. Likewise, high self-efficacy can support the rehabilitation process by improving adherence [55]. 

Personality characteristics are an additional pillar in the Model of Stress and Athletic Injury [21]. Such personality characteristics include, for example, locus of control, sense of coherence, hardiness, and competitive trait anxiety, which influence the stress response of athletes and thus the risk of injury. In a recent meta-analysis by Ivarsson et al. [15], however, personality factors had a negligible relationship with injury rates. Yet, depending on the outcome and measurement tools used, the study results are mixed with some evidence underlining the influence of (competitive) anxiety on the injury risk of athletes [56,57,58]. The results are also mixed for locus of control: there are studies that demonstrate an association between locus of control and (risk of) sports injuries [59,60] and studies that find no evidence for this [61]. Hardiness and the related concept of resilience are especially important after injury, as they can make it easier to deal with sports injuries, with people being more resilient and showing more hardiness being better able to cope with an injury and staying motivated during the rehabilitation process [62]. However, hardiness has also been investigated prior to an injury hypothesizing that people being hardier sustain less injuries [63]. Furthermore, sense of coherence has been investigated scarcely in relation to sports injuries [21]. However, in a cross-sectional study, sense of coherence was negatively associated with lay-offs due to overuse injuries in young female athletes [64]. Additionally, sense of coherence seems to play an important role in maintaining mental health after an injury [31]. In addition to the personality factors stated in the Model of Stress and Athletic Injury, several others have been investigated and related to injury risk and occurrence in the past years. For example, athletic identity and excessive effort seem to be related to injury risk with higher athletic identity [65] and higher excessive effort [28,66,67] being risk factors for injury. 

In summary, the present study had two aims: the first aim was to examine whether distinct clusters of injury patterns can be identified by using various injury characteristics (e.g., frequency, severity, treatment); the second aim was to compare the obtained clusters of psychosocial variables suggested by previous research and the Model of Stress and Athletic Injury [21]. 

## 2. Materials and Methods

### 2.1. Study Design

The presented data was collected in a cross-sectional online study conducted with SoSci-Survey [68]. The recruitment of athletes and data collection took place in January and February 2022 by contacting sport students at universities in Germany and Austria via e-mail through their respective academic offices. Additionally, athletes from various team and individual sports throughout Germany were invited to participate in the study by e-mail via their respective clubs or sports associations as well as social media channels. The inclusion criteria were being athletes with an age of 16 to 40. Additionally, they should have had a sports injury in the past or be currently suffering from a sports injury. Participation was voluntary. Sport students received a compensation of EUR 15 for participating in the study. All other participants could take part in a raffle and win a total of 20 prizes worth EUR 25 each. All participants gave written informed consent and agreed with the General Data Protection Regulation before completing several questionnaires with a total length of 30–40 min. The online study included questions about socio-demographic variables as well as sports- and injury-related questions and questionnaires assessing several psychosocial variables (for more details, see the “Method” section). Approval for the study was granted by the local Review Board of the Institute for Psychology of Johannes Gutenberg-University in Mainz and follows the guidelines of the Declaration of Helsinki [69].

### 2.2. Power Analysis

The first step and aim of the study was to identify homogeneous clusters of injury patterns based on different injury characteristics. That is why we based our power analysis on requirements for the cluster analysis. According to Dalmaijer et al. [70], there should be at least n = 20 per included indicator submitted to the cluster analysis. As we included six such indicators, *N* = 120 was the minimum number of participants for conducting a cluster analysis. Calculation of sample size for the analyses of differences between clusters was more difficult as we did not know the final number of clusters beforehand. A priori sample size calculations with G*Power [71] with a medium effect size (f^2^ = 0.15), an error probability of α = 0.05, a power ß = 0.80, group numbers between 2 and 6, and number of response variables between 2 and 10 resulted in a required sample size of *N* = 36 to *N* = 118.

### 2.3. Sample

In total, we collected data from 427 participants. For our analyses, however, we excluded participants not completing the entire survey (n = 195). Additionally, 19 participants were excluded from data analyses because of inconsistencies in the responses or discrepancies with the initially stated exclusion criteria in the instructions of the survey (e.g., no injury, older than the age limit). The final sample included in the analyses consisted of *N* = 213 athletes. Table 2 provides an overview of the demographic data and characteristics of the final sample. Most of participants were students (87.79%) enrolled in a bachelor’s or master’s degree in sports studies (70.47%), with the remaining proportion (12.21%) being either in full-time training, unemployed, full-/part-time, self-employed, or civil servant.

### 2.4. Measures

In the following, measures relevant to this study are specified (for an overview of all measures see Supplement S1). All questionnaires were presented in German. Mean, standard deviation, 95% confidence interval, and the scales’ or subscales’ Cronbach’s α of the current sample are depicted in Table 3.

#### 2.4.1. Injury-Related Questions

Before starting the questionnaire, participants were given the following definition of injuries: “In this study, we define injury as events that either required medical treatment or resulted in an interruption of your athletic activity (e.g., termination of training/competition/athletic testing) of at least 1 day”. Injury characteristics, which were included in the analyses, are described in detail below.

***Injury frequency.*** Participants were asked to list the number of injuries they had sustained in the last 12 months in: (1) training/ practical courses; (2) in competition; (3) during a study-related examination. Numbers were added to a general frequency score.

***Injury status.*** Participants were asked to indicate whether they were currently injured due to sport activities. They could either answer “yes” or “no”. If they answered “yes”, they were asked several follow-up questions regarding the current injury. If they answered “no”, they were asked several follow-up questions regarding a past injury.

***Injury severity.*** Severity was measured via constraints in sports activities with the following five answer options: (1) “I have no restrictions on my sports activities”; (2) “I have hardly any restrictions on my sports activities”; (3) “I will probably have to interrupt my sports activities for 1–7 days due to the injury”; (4) “I will probably have to interrupt my sports activity for 8–21 days due to the injury”; and (5) “I will probably have to interrupt my sports activity for >21 days due to the injury”.

***Medical treatment.*** Participants indicated whether they are (current injury) or have been (past injury) receiving medical treatment for their injury with “yes” or “no”. 

***Rehabilitation.*** To assess rehabilitation measures, participants were either asked whether they are currently in a rehabilitation program (including physical therapy/ outpatient rehab) for their injury (current injury) or whether they have been in a rehabilitation program (including physical therapy/outpatient rehab) for their injury (past injury). Answer options were “yes” and “no”. If they answered “yes”, an additional free-text field appeared where they could further describe the rehabilitation program. 

***Chronicity*.** Participants were asked whether they suffered from a chronic injury caused by their sports activities. Answer options were “yes” and “no”. If they answered “yes”, an additional free-text field appeared where they could further describe the injury. 

#### 2.4.2. Psychosocial Factors

***Athletic Identity.*** To measure the athletic identity of participants, the German version of the Athletic Identity Measurement Scale (AIMS-D) [72] was used. The AIMS-D contains three subscales: social identity (3 items), exclusivity (2 items), and negative affectivity (items). We formed a global mean of scale. Participants indicated their agreement on a 7-point Likert scale ranging from 1 (=strongly disagree) to 7 (=strongly agree). An example item is “I consider myself an athlete”.

***Excessive Effort.*** Excessive effort was assessed with the German version of the Excessive Effort in Sport Scale (EESS) [28,73]. The inventory consists of 18 items and contains statements from athletes about various behaviors and experiences in sports (e.g., “I wish to be recognized for my commitment in sports”.). Items are rated on a 5-point Likert scale (1 = not at all true; 5 = completely true).

***Locus of Control.*** The Internal-External Locus of Control Short Scale–4 (IE-4) [29] containing four items was used to measure control beliefs of participants. They rate two items each for internal and external control beliefs on a 5-point Likert scale (1 = strongly disagree, 5 = strongly agree) and state their agreement with statements such as “I am in control of my life”. 

***Resilience.*** The German version of the Brief Resilience Scale (BRS) [30] was used to assess participants’ resilience. Participants state their agreement with statements such as “I tend to bounce back quickly after hard times”. on a 5-point Likert scale (1 = strongly disagree, 5 = strongly agree). 

***Sense of Coherence.*** To measure the sense of coherence of participants, the Sense of Coherence Leipzig Short Scale (SOC-L9) [74] was used. The SOC-L9 consists of nine items and contains three subscales (comprehensibility, manageability, and meaningfulness) that should not be interpreted in this short version. Participants indicated their agreement on a 7-point Likert scale ranging from 1 to 7. Depending on the respective item, the scale anchors varied. An example statement is “How often are your feelings and ideas all mixed up?”.

***Competition anxiety.*** To assess sport-specific anxiety, we applied the Competition-Anxiety-Inventory (Wettkampf-Angst-Inventar Trait; WAI-T) [75]. It comprises twelve items, four of which are assigned to each of the following subscales: somatic anxiety, concerns, and concentration disruption. Participants rate the items on a 4-point Likert scale (1 = not at all, 4 = very much). An example item is “Before competitions I feel nervous”.

***Fear of (re)injury.*** The German Version of the Tampa Scale of Kinesiophobia (TSK-GV) [76] was used to measure fear of movement/(re)injury. Participants rated on a 4-point Likert scale (1 = strongly disagree, 4 = strongly agree) items like “I am afraid of possibly hurting myself when I play sports”. 

***Life Events.*** Stressors were measures with the Social Readjustment Rating Scale (SRRS) [34] consisting of 43 items. Participants were asked whether a specific event (e.g., divorce, job loss) has occurred in the past year or is expected to occur soon. They could either choose “yes” or “no”. Each event is assigned an impact score (e.g., death of spouse = 100, vacation = 13) which are summed up to a total score. According to the authors, a total score of 150 or less equals a low level of stress, whereas a score between 150–299 is considered a moderate level, and a score of 300 or above as a high level of stress. 

***Stress.*** The perceived stress of participants was measured via the Perceived Stress Questionnaire–20 (PSQ-20) [35], including the subscales: demands (5 items), joy (5 items), tension (5 items), and worries (5 items). In the current study, we formed a global mean of scale. For each item, participants were asked to indicate on a scale from 1 (=almost never) to 4 (=most of the time) to what extent they agreed with statements such as “You fear not being able to achieve your goals”.

***Self-compassion.*** The twelve-item Self-Compassion Scale short version (SCS-D short version) [36,77] was used to measure self-compassion. The SCS-D short version is based on a 5-point Likert scale ranging from 1 (=very rarely) to 5 (=very often). An example statement is “I try to see my mistakes as part of human nature”.

***Mindfulness.*** To assess mindfulness, the short version of the Mindful Attention and Awareness Scale (MAAS-short) [37] was used. It contains ten items (e.g., “I notice how I do things without paying attention to them”.) and is rated on a 6-point Likert scale (1 = almost always, 6 = almost never).

***Social support.*** To measure social support, the short version of the Social Support Questionnaire (F-SozU K-14) [78] was used. The questionnaire contains 14 items and is rated on a 5-point Likert scale (1 = strongly disagree, 5 = strongly agree). An example statement is “There are people who take me as I am without restriction”.

***Self-efficacy.*** Sports-related self-efficacy was assessed with the Exercise Self-Efficacy Scale (ESES) [39]. It queries the belief that athletes can stick to training, even under unfavorable circumstances. The scale consists of 12 items (e.g., “I am confident that I can still perform a planned sports activity even when I am tired”.) and is rated on a 7-point Likert scale (1 = not sure at all, 7 = for certain).

***Coping.*** The Coping Orientation to Problems Experienced Inventory (Brief-COPE) [40] was used to assess adaptive (α = 0.79) and maladaptive (α = 0.65) coping. The questionnaire consists of 28 items, which are answered on a 4-point Likert scale (1 = not at all, 4 = very much). An example statement for adaptive coping is “I focused on changing something about my situation”.

### 2.5. Data Preparation and Screening

One participant indicated an invalid age, but the correct age could be reconstructed from other information replacing the original value. During data processing, the items in question were recoded and combined to form a scale mean or sum score, respectively. To investigate whether the data was normally distributed, graphical (histograms, QQ plots) and statistical (Shapiro–Wilk test) approaches were used. As the Shapiro–Wilk test was significant (*p* < 0.05) for all other variables than fear of reinjury, self-compassion, adaptive coping, excessive effort, and mindfulness, this indicates a violation of the normal distribution assumption in most cases. Therefore, multivariate normality was also not given. 

### 2.6. Data Analyses

Data analyses were conducted with R [79] and RStudio [80]. An error probability of α = 0.05 was set a priori for all interference statistics calculations. All analysis scripts necessary to replicate the analysis of the present study are available online: osf.io/ctb9n (accessed on 17 November 2023).

#### 2.6.1. Cluster Analyses

Since the data was mixed (i.e., binary, interval), the function “daisy” [81] from the package *cluster* with the metric “gower” [82] was used to conduct cluster analyses. It computes all the pairwise dissimilarities (distances) between observations in the data set and uses the general dissimilarity coefficient of Gower [82]. Further going, the function “pam” from the package *cluster* was used. It is an algorithm which enables partitioning (clustering) of the data into k clusters “around medoids” and is known to be a more robust version of K-means. Cluster choice was based on theoretical and content-based considerations as well as statistical criteria. From all collected injury-related variables we solely included injury frequency, current injury status, severity, medical treatment, rehabilitation measures and chronicity as these injury-related variables could be relatively objectively and easily used on a screening checklist in later studies. In contrast, cause of injury and drug treatment have shown to be rather subjective and inconclusive. For example, causes such as “accident while landing a megaloop (kiting)”, “twisted while warming up in karate during an evasive maneuver”, “while skiing in a turn—ski twisted and fell” or “twisted on an awkwardly created edge” could be attributed to both, either external or internal circumstances or even a combination of both which participants could not choose. Regarding drug treatment, we could not distinguish between self-medication and prescription by medical stuff. Setting and date were excluded as we do not want to distinguish participants on these variables. Regaining previous performance level and duration of recovery was excluded because only participants with a past injury could answer these questions. Similar applies to the consequences of the injury. Currently injured participants may estimate the consequences based on their current knowledge, but participants may recover faster as expected or be confronted with setbacks in the rehabilitation process influencing the consequences. Dendrograms, elbow, and silhouette plots [83] were conducted with various cluster numbers. A silhouette coefficient of >0.70 was considered strong and a coefficient of >0.50 as a reasonable structure of the data [81].

#### 2.6.2. Differences in Psychosocial Variables

Building up on the three pillars of the Model of Stress and Athletic Injury [21], three MANOVAs were planned. The calculation of a MANOVA is appropriate when the dependent variables are correlated with each other since shared variance is included in the calculations of differences [84]. To check for interrelation between psychosocial variables and due to the violation of the normal distribution [84], we calculated Spearman correlations of all psychosocial variables. These are shown in Table 4. If the MANOVAs were significant, several univariate analyses of variance (ANOVA) and post-hoc t-tests were performed for the individual dependent variables to capture the respective group differences. An eta-squared of 0.01 usually equals a small effect, 0.06 represents a medium effect, and 0.14 can be considered as a large effect [85]. 

## 3. Results

### 3.1. Cluster Analysis

Although it is recommended that the silhouette coefficient should be >0.50 to assume a reasonable data structure [81], we finally decided on the 3-cluster solution based on content-wise considerations. As Table 5 shows, the average silhouette coefficient increases slowly with the number of clusters, being for the first time >0.50 at eight clusters; however, even for that solution, the coefficient ranges from 0.15 to 0.73. As for the eight or more cluster solutions, the smallest cluster contains only twelve or less participants; therefore, we discarded these solutions in favor of more balanced sample sizes for each cluster and in favor of a more suitable interpretation of the clusters.

Descriptive values of the 3-cluster solution are presented in Table 6. Cluster 1 is characterized by a majority of currently injured (79.63%) athletes suffering from chronic injuries (72.22%) and being treated medically (90.74%). Cluster 2 is characterized by athletes seeking almost no treatment (11.29%) or utilizing rehabilitation (8.06%). Comparatively, Cluster 3 is characterized by a lower injury frequency (*M* = 1.57) but higher injury severity (*M* = 4.57), as well as medical treatment (96.91%) and rehabilitation measures (81.44%) reported by almost all athletes.

To check whether the classification of the clusters was influenced by differences in demographic or sports-related aspects, we performed χ^2^ tests and ANOVAs (Table 7). The clusters did not differ significantly in age, gender, sports type (individual vs. team), training sessions per week in the respective sport and in total, training hours per week, years of exercising this sport, performance level, participating in competitions, competition level, squad status, membership of a national team, or utilizing mental training (*p* > 0.05). 

### 3.2. Results of MANOVAs

Prior to calculating the MANOVAs and ANOVAs, the data were examined for outliers, normal distribution, and variance homogeneity (Field et al., 2012). Variance homogeneity of groups (Cluster 1, 2, and 3) was given for all variables (Levene tests: *p* > 0.05) except excessive effort, *F*(2, 210) = 4.63, *p* = 0.011. However, homogeneity of covariance matrices were given in all planned MANOVAs (Box’s M Test: *p* > 0.05). Although it is argued that (M)ANOVAs are relatively robust for non-normally distributed data [84,86,87], the data should be viewed in the light of the previously reported outliers and violation of the univariate and multivariate normal distributions. 

Cluster allocation was used to assess between-subject factors. Dependent variables were summarized to represent the three pillars: personality factors (i.e., athletic identity, excessive effort, control beliefs, resilience, sense of coherence, competition anxiety, fear of reinjury), history of stressors (i.e., life events and perceived stress in the last two years), and coping resources (i.e., self-compassion, mindfulness, social support, sport-specific self-efficacy, and coping styles). There was a significant main effect of group for personality (*F*(2, 170) = 2.01, *p* = 0.007, η_p_² = 0.11), history of stressors (*F*(2, 210) = 2.76, *p* = 0.028, η_p_² = 0.03), and coping resources (*F*(2, 210) = 2.53, *p* = 0.003, η_p_² = 0.07).

### 3.3. Results of ANOVAs and Post-Hoc Analyses

As shown in Table 8, the ANOVAs revealed significant group differences on the following psychosocial factors: excessive effort and sense of coherence (personality), perceived stress (history of stressors), as well as self-compassion, mindfulness, and sport-specific self-efficacy (coping resources). Regarding means and standard deviations (Table 7), Cluster 1 (currently injured/chronic injuries) always had higher (i.e., perceived stress, excessive effort, sport-specific self-efficacy) or lower scores (i.e., self-compassion, mindfulness, sense of coherence) than the other clusters.

Bonferroni–Holm-corrected [88] post-hoc tests revealed significant differences between Cluster 1 and Cluster 3 in sense of coherence (*p* = 0.013), perceived stress (*p* = 0.011), mindfulness (*p* = 0.034), and self-compassion (*p* = 0.002). Cluster 1 perceived a lower sense of coherence as well as higher stress and assessed themselves as less mindful and less self-compassionate than Cluster 3. Additionally, there was a significant difference between Cluster 1 and Cluster 2 in self-compassion (*p* = 0.001) and sport-specific self-efficacy (*p* = 0.021), with lower (self-compassion) and higher (self-efficacy) values in Cluster 1. No significant differences were found between Cluster 2 and 3. Difference in excessive effort was non-significant (*p* > 0.05). 

## 4. Discussion

The present study aimed to examine whether currently or previously injured athletes can be clustered into different injury patterns based on treatment information (i.e., medical treatment and rehabilitation measures) and injury characteristics (i.e., current injury status, frequency, severity, and chronicity) and if these distinct patterns differ with respect to relevant psychosocial variables. Cluster analysis revealed a three-cluster solution, which differed substantially in treatment (Cluster 1 receiving mostly medical treatment, Cluster 2 seeking barely any treatment, and Cluster 3 utilizing medical treatment and rehabilitation) and chronicity (Cluster 1 high rates of chronic injuries, Cluster 2 and 3 with low levels of chronicity). The clusters did not differ in any demographics or sports-related information. However, comparing the three clusters with respect to the three pillars of the Model of Stress and Athletic Injury [21] (personality factors, history of stressors, and coping resources) revealed differences in all three areas with Cluster 1 experiencing a significantly higher stress load, reporting higher excessive effort and sport-specific self-efficacy, as well as less coping resources (sense of coherence, self-compassion, and mindfulness) than the other two clusters. 

Previous research about the psychology of sports injuries has investigated various causes and predictors of and reactions to sports injuries. However, up to date, they have mostly focused only on one characteristic of injuries (e.g., frequency) or single selected predictors. To the best of our knowledge, this study is the first one to assess several injury characteristics and use a data-driven approach to cluster athletes with sports injuries into different categories. In doing so, we aimed to address the complexity of sports injuries and possibly derive a better interpretation of injury data [18]. For example, the frequency of injuries has been an often-used measurement when using psychosocial factors to predict injuries [23,89]. However, in our data, the frequency of injuries within the past 12 months was not a major contributor to cluster choice, whereas low or high level of treatments and current injury status combined with chronicity distinguished the clusters. The latter one is especially important as the cluster of currently and chronically injured is the most burdened group and the one with the lowest coping resources. Surprisingly, there is almost no research about psychosocial factors and chronic injuries with only one study investigating 280 athletes (42% of them chronically injured) and coming to the conclusion that chronically injured athletes experience higher levels of distress [90]. One possible explanation for the elevated levels of perceived stress in the first cluster is the fact that most athletes in Cluster 1 are currently injured, and probably must deal with pain and the consequences of the injury (e.g., missed competition, rehab). Another explanation is that the chronicity of the injuries leads to elevated levels of perceived stress as diagnostics and treatments of chronic injuries are often more complex than of acute injuries. An explanation for lower levels of sense of coherence in athletes of the first cluster is that athletes sustaining a chronic injury and having to deal with a prolonged diagnostic and treatment process may perceive their situation as less comprehensible, manageable, and meaningful as athletes who have a specific diagnosis and a specific rehabilitation plan even though the injury might go along with a sports break of several weeks or months. Additionally, Cluster 2 and 3 consist of less currently injured athletes. Possibly athletes in Cluster 2 and 3 have found and used coping resources and mechanisms to deal with their injury so that they experience their current situation as more comprehensible, manageable, and meaningful than the currently injured athletes who still must go through this process. 

Investigating injuries in ballet dancers and trying to explain the occurrence of chronic injuries, Hamilton et al. [91] (p. 267) state that “for the elite ballet dancer, the very qualities that are necessary in the individual’s continual drive toward physical perfection can also lead to a history of chronic injuries if carried to an extreme”. Bringing this quote together with our results, a high excessive effort and going beyond one’s own boundaries, maybe without noticing or acknowledging one’s limits due to low levels of mindfulness and self-compassion, could be risk factors for chronic injuries and explain the present results.

The result of higher sport-specific self-efficacy values in the cluster of currently and chronically injured fits into the results that athletes with higher self-efficacy tend to seek greater challenges [92], possibly elevating the risk for getting injured. When assessing self-efficacy, we used a sport-specific self-efficacy scale which encompassed items such as “I am confident that I can still perform a planned sports activity even if I am tired”. In that regard, higher sports-related self-efficacy might reflect a tendency of chronically injured athletes (Cluster 1) to participate in training activities despite their injury (e.g., to prevent training backlog), whereas rather healthy athletes (Cluster 2) seeking no treatment respect their injury-related impairments (e.g., to be tired). However, this interpretation is rather speculative as our results are based on cross-sectional data and thus, no causal conclusions are possible. Further research on the specific group of chronically injured athletes is needed to obtain a clearer picture of the relation between psychosocial factors and chronic injuries, and future longitudinal studies in injured athletes might follow-up on that question. 

Regarding the non-significant differences found in the present investigation, there are various possible explanations. First, the violation of normal distribution in most collected variables, along with inspection of data, indicates the presence of both floor effects (e.g., external control belief) and ceiling effects (e.g., social support, internal control belief, athletic identity). These effects limit variance in our dataset, which contained a large proportion of sports students, which usually have an adequate social network. Nevertheless, descriptive trends appear to support the previously mentioned conclusions. For example, although non-significant, Cluster 1 tends to exhibit a higher athletic identity and lower levels of resilience than the other two clusters. Attempting to explain these trends, they could mean that athletes with chronic injuries tend to perceive themselves as athletes, investing excessive effort into training and participating in training activities despite their injury because they perceive themselves as less capable of dealing with setbacks or bounce back after stress (resilience). However, future research specifically focusing on athletes with chronic injuries is needed. Another possible explanation for the non-significant results compared to previous studies is that prior research linked psychosocial factors to individual injury characteristics (e.g., anxiety and injury incidence rate, [58]). We did not analyze whether psychosocial factors differ with respect to single specific injury characteristics (e.g., currently injured vs. currently not injured), although it is conceivable that, for example, currently injured athletes experience more fear of (re-)injury than currently non-injured athletes. Athletes who have gone through more injuries within the past 12 months may show higher levels of resilience due to accumulated knowledge on how to successfully deal with injuries compared to athletes with little or no injuries within the last 12 months [93]. 

### 4.1. Limitations and Further Research

The results of the present study must be viewed in light of some limitations. First, cluster fit was not perfect and injuries as well as sample characteristics (e.g., specific sport type) were heterogenous. Although most characteristics of the sample such as sport types, training hours per week, and so forth, seem to be equally distributed over the different clusters, some criteria had generally low figures of representatives (e.g., two professionals compared to eighty-five amateurs) limiting the generalizability of the results and the informative value of the data. The distinction, for example, between performance groups and in-depth examinations might be interesting, as the consequences of sports injuries may be more severe and life-changing for high performance athletes than for amateurs. However, usually medical treatment and rehabilitation measures are more accessible to high performance athletes. Further research could therefore focus on specific sport types, age, or performance groups, to investigate the presented cluster solutions in-depth and possibly replicate them to gain a deeper understanding of possible injury patterns. 

Second, assessing injury and its broader context can be extended and refined. Thus, although we included several injury characteristics, we did not assess whether the injuries were re-injuries or subsequent injuries of relieving postures or other injuries. Additionally, although we assessed chronicity, we did not assess whether the reported injuries were caused by trauma or caused by overuse. This differentiation will be important for future studies as according to Gledhill and Forsdyke [94], overuse injuries are underrepresented and the main focus of injury research has been on acute and traumatic injuries, even though approximately 30–40% of sports injuries can be classified as overuse injuries [19]. In a study with gymnasts, it was even around 64% [95]. We also did not assess whether participants had to be operated on and whether the participants did experience pain even though they had no specific injury diagnosed. Moreover, the severity of injuries had kind of a ceiling effect, as most participants reported taking a break longer than 21 days. In further research, severity should be classified in more steps to differentiate between interruptions around 21 days (e.g., due to ligament stretch) or up to 6 months or more (e.g., due to cruciate ligament tear) or even be classified differently. One possibility would be to assess functionality despite the injury instead or in combination with time loss due to injury. In addition, the present study included only athletes who were currently injured or had been injured in the past, whereas athletes without injury were not included. However, as sports injuries are ubiquitous, it might be hard to find many athletes who have never sustained any kind of sports injury. Other factors such as consequences, drug treatment, recovery time, or cause were also not included in the present cluster analysis for various reasons. One limitation was that all measures were based on self-report which, on the one hand, comes along with the well-known limitations (e.g., psychometric properties, response biases) but on the other hand also led to the exclusion of cause and drug treatment. Future studies could collect data from medical staff to gain specific diagnoses or differentiate between self- or prescribed treatment. Generally, combining self- and third-party reports could increase the accuracy of assessing sports injuries. Consequences (e.g., career-ending, financial losses) and recovery time of sports injuries can be included in the future to better define injury patterns by using longitudinal designs. 

Longitudinal designs can also counteract another limitation of this study: due to the cross-sectional design of the study, no causal conclusions can be drawn. Further research should therefore examine these cluster groups and psychosocial mechanisms in a prospective longitudinal design with repeated measures [96] to capture the dynamics, interactivity, and complexity of sports injuries and psychosocial processes. For example, it would be interesting to investigate whether these clusters are stable over time or if participants change the cluster during, for example, healing processes. Additionally, the causal mechanisms and relationships of the clusters and psychosocial variables should be investigated to determine the relevant psychosocial factors pre- and post-injury, to develop effective interventions and for example, to answer questions like whether athletes in Cluster 1 are generally less self-compassionate, less mindful, and experience more stress load than the other clusters, and therefore become chronically injured or vice versa. Longitudinal designs could also help to distinguish whether actuality, chronicity, or both were the driving force of differences between the clusters. 

Finally, the present study focused mainly on the individual level. However, injuries—especially overuse and probably also chronic injuries—seldomly occur isolated but rather in a complex interaction between the situation (e.g., cup finals vs. beginning of the season), individual factors (e.g., injury history, personality), team and coach (e.g., culture of pain), as well as the club and federation (e.g., interest in short-term gain) [97,98]. Sport sociocultural norms and rules, following a specific sports ethics [99] usually connected with a “culture of pain”, playing through pain and making sacrifices, and a poor coach–athlete relationship [98] may elevate the risk of sustaining an injury. Therefore, further research should encompass not only intrapersonal but also interpersonal factors. Along with the sociocultural norms of the specific sport, a general problem in injury research is that in some sports and some cultures, minor injuries needing no or low treatment are not even viewed as injuries and often are not reported at all. Eventually, this problem can be addressed in a repeated measures design with more frequent assessment points and confidentiality measures. Moreover, addressing the complexity and interactivity of sports injuries, network models as used by Hill and Den Hartigh [100] can be useful. Especially, having a look at changes in physiological and psychological factors and changes in these networks can help to predict the occurrence of sports injuries in general and different forms of injuries specifically.

### 4.2. Practical Implications

The findings of our study implicate that distinct injury patterns relate differently to psychosocial factors. Practitioners working with injured athletes should therefore examine closely the individual circumstances of their clients. Our results suggest that currently and chronically injured athletes are an especially burdened group, and this work sheds light on factors to address in interventions. Based on our results, we cannot distinguish if, for example, self-compassion is important in preventing injuries, in dealing with injuries, or both. However, building up on the results presented, it seems to be important to improve stress management, foster mindfulness and self-compassion, and address problematic influences of high excessive effort combined with high self-efficacy beliefs, particularly with athletes sustaining chronic injuries or even a history of chronic injury. 

## 5. Conclusions

Sports injuries are ubiquitous. With their potentially far-reaching consequences, it is necessary to develop and implement effective measures to prevent and reduce injury occurrence and facilitate the management of sports injuries. The present study highlights the importance of paying attention to several characteristics of injuries that form specific injury patterns and differently relate to psychosocial factors. Further research should refine the classification of sports injuries and replicate clusters as well as cluster differences. In the long run, target-specific interventions for those being especially at risk of injury and/or burdened by injury should be developed.

## Figures and Tables

**Table 2:** Demographics and sport-related information: frequency (n), percentage (%), mean (M), standard deviation (SD).

	n	%	M	SD
Age	–	–	22.77	3.57
Sex	213	100	–	–
Female	115	53.99	–	–
Male	95	44.60	–	–
Non-binary	3	1.41	–	–
Training sessions per week in main sports	–	–	3.50	1.98
Trainings sessions per week in total	–	–	6.27	2.93
Hours of sport per week	–	–	10.94	5.80
Number of years practicing the respective sport	–	–	12.30	5.62
Individual sports	94	43.66	–	–
Fitness	11	5.16	–	–
Combat sports	10	4.69	–	–
Running	7	3.29	–	–
Athletics	19	8.92	–	–
Tennis	8	3.76	–	–
Triathlon	7	3.29	–	–
Gymnastics	10	4.69	–	–
Other *	22	10.33	–	–
Team sports	119	56.33	–	–
Basketball	10	4.69	–	–
Soccer	60	28.17	–	–
Handball	26	12.21	–	–
Volleyball	11	5.16	–	–
Other *	12	5.63	–	–
Participation in competitions				
Regularly	141	66.20	–	–
Now and then	34	15.96	–	–
Very rarely	18	8.45	–	–
No competitions	20	9.39	–	–
Performance level				
Professional level	2	1.30	–	–
High-performance level	18	7.83	–	–
Competitive level	108	48.70	–	–
Amateur level	85	42.17	–	–
Competition level				
International	18	69.27	–	–
National	45	21.95	–	–
Regional	142	8.78	–	–
Squad level				
Highest national level or Olympic squad	13	6.10	–	–
Second highest national level or perspective or supplementary squad	13	6.10	–	–
Third highest national level or junior squad	14	6.57	–	–
Fourth highest national level or national squad	35	16.43	–	–
Other lower competition level or other squad status	86	40.38	–	–
No competition level or squad status	52	24.41	–	–
National team				
Yes	24	11.27	–	–
No	189	88.73	–	–
Mental training				
Yes	32	15.02	–	–
No	181	84.98	–	–

Note. * Including sport types that were stated by 5 or less participants (bike sports = 5; cheerleading n = 2; fist ball n = 1; hockey n = 2; inline-speedskating n = 1; climbing n = 2; lacrosse n = 3; swimming n = 5; wheel gymnastics n = 1; table tennis n = 2; water sports n = 1; multiple sports n = 4).

**Table 3:** Mean (M), standard deviation (SD), 95% confidence interval (95% CI), and Cronbach’s alpha (α) of psychosocial variables.

	Questionnaire	M	SD	95% CI	α
**Personality factors**					
Athletic identity	AIMS-D	5.18	1.00	[5.04; 5.31]	0.82
Excessive effort	EESS	3.20	0.50	[3.13; 3.26]	0.82
Internal control belief	IE-4	4.19	0.66	[4.11; 4.28]	0.69
External control belief	IE-4	2.31	0.72	[2.21; 2.41]	0.49
Resilience	BRS	3.39	0.70	[3.29; 3.48]	0.80
Sense of coherence ^a^	SOC-L9	4.87	0.98	[4.72; 5.01]	0.86
Competition anxiety	WAI-T				
Somatic anxiety		2.60	0.82	[2.49; 2.71]	0.85
Concentration disruption		1.72	0.57	[1.64; 1.80]	0.91
Concerns		2.52	0.76	[2.42; 2.62]	0.85
Fear of (re-)injury	TSK-GV	2.17	0.43	[2.11; 2.23]	0.66
**History of stressors**					
Life events	SRRS	208.15	86.15	[196.52; 219.79]	0.71
Stress	PSQ-20	2.18	0.48	[2.12; 2.25]	0.92
**Coping resources**					
Self-compassion	SCS-D short	3.04	0.66	[2.95; 3.13]	0.86
Mindfulness	MAAS	3.94	0.69	[3.84; 4.03]	0.84
Social support	F-SozU K-14	4.38	0.65	[4.29; 4.64]	0.93
Sport-specific self-efficacy	ESES	5.62	0.84	[5.51; 5.74]	0.84
Maladaptive coping	Brief-COPE	2.07	0.37	[2.02; 2.12]	0.65
Adaptive coping	Brief-COPE	2.60	0.42	[2.54; 2.65]	0.79

Note. ^a^ n = 173.

**Table 4:** Spearman correlations of psychosocial variables.

	1	2	3	4	5	6	7	8	9	10	11	12	13	14	15	16	17
**Personality factors**																	
1. Internal control belief																	
2. External control belief	−0.43 ***																
3. Excessive effort	−0.09	0.11															
4. Athletic identity	0.10	−0.05	0.35 ***														
5. Resilience	0.38 ***	−0.38 ***	−0.08	0.13													
6. CA–Somatic anxiety	−0.19	0.20	0.04	−0.04	−0.19												
7. CA–Concentration disruption	−0.30 **	0.22	0.03	−0.11	−0.28 **	0.22											
8. CA–Concerns	−0.27 **	0.25 *	0.16	−0.06	−0.29 **	0.52 ***	0.32 ***										
9. Fear of injury	−0.16	0.22	−0.02	−0.10	−0.19	0.21	0.13	0.11									
10. Sense of coherence	0.53 ***	−0.44 ***	−0.14	0.01	0.48 ***	−0.21	−0.36 ***	−0.36 **	−0.12								
**History of stressors**																	
11. Life events	−0.05	0.01	0.05	−0.02	0.05	0.11	−0.06	0.02	0.10	−0.08							
12. Stress	−0.48 **	0.39 ***	0.25 *	−0.05	−0.43 ***	0.30 ***	0.35 ***	0.50 ***	0.24 *	−0.68 ***	0.16						
**Coping resources**																	
13. Sport-specific self-efficacy	0.24 *	−0.15	0.12	0.45 ***	0.27 **	−0.02	−0.22	−0.14	−0.22	0.12	0.00	−0.07					
14. Self-compassion	0.37 **	−0.28 **	−0.30 ***	−0.14	0.41 ***	−0.29**	−0.22	−0.40 ***	−0.13	0.58 ***	−0.16	−0.60 ***	−0.03				
15. Social support	0.41 ***	−0.26 *	−0.13	0.01	0.26 *	−0.03	−0.19	−0.17	−0.11	0.52 ***	−0.16	−0.48 ***	0.18	0.33 ***			
16. Adaptive coping	0.37 ***	−0.16	−0.09	−0.02	0.30 ***	0.01	−0.14	−0.14	−0.00	0.51 ***	−0.06	−0.37 ***	0.10	0.48 ***	0.37 ***		
17. Maladaptive coping	−0.19	0.26 *	0.12	0.09	−0.22	0.21	0.25 *	0.27 **	0.16	−0.28	−0.06	0.23	−0.11	−0.27 **	−0.14	0.00	
18. Mindfulness	0.29 **	−0.24 *	−0.20	−0.03	0.23	−0.12	−0.32 ***	−0.31 ***	−0.03	0.58 ***	−0.08	−0.47 ***	0.02	0.44 ***	0.23	0.36***	−0.15

Note. CA = competition anxiety; Bonferroni–Holm-adjusted *p*-values, * *p* < 0.05; ** *p* < 0.01; *** *p* < 0.001.

**Table 5:** Descriptive values of the silhouette coefficient.

Number of Clusters									
	2	3	4	5	6	7	8	9	10
Average silhouette width	0.36	0.40	0.41	0.45	0.46	0.48	0.51	0.56	0.61
Silhouette width range	0.31; 0.40	0.25; 0.46	0.16; 0.53	0.17; 0.60	0.25; 0.76	0.17; 0.75	0.15; 0.73	0.23; 0.78	0.46; 0.78

**Table 6:** Injury-related information of the total sample and respective clusters: mean (M)/frequency (n), standard deviation (SD)/percentage (%).

	n	Currently Injured	Frequency ^a^	Severity ^b^	Medical Treatment	Rehabilitation	Chronicity
All	213	36.09%	1.94 (1.79)	3.87 (1.18)	70.42%	44.60%	27.83%
Cluster 1	54	79.63%	2.33 (2.11)	3.41 (1.37)	90.74%	20.37%	72.22%
Cluster 2	62	19.35%	2.19 (1.85)	3.18 (0.88)	11.29%	8.06%	11.29%
Cluster 3	97	23.71%	1.57 (1.48)	4.57 (0.80)	96.91%	81.44%	13.40%

Note. ^a^ Range [0; 12], ^b^ Range [1; 5].

**Table 7:** Demographic and sports-related data of respective clusters: mean (M), standard deviation (SD), frequency (n), and percentage (%); Results of ANOVAs and χ^2^ tests.

Outcome	Cluster 1		Cluster 2		Cluster 3		ANOVA between Group		
	M	SD	M	SD	M	SD	*F*(2, 210)	*p*	η_p_²
Age	23.35	3.82	22.56	3.43	22.59	3.51	0.95	0.390	0.00
Training sessions per week in main sports	3.96	2.39	3.58	1.88	3.28	1.78	2.10	0.125	0.02
Training sessions per week in total	6.65	3.06	5.94	2.61	6.27	3.04	0.85	0.427	0.00
Hours of sport per week	12.24	7.54	11.11	6.04	10.3	4.25	1.97	0.142	0.02
Number of years practicing the respective sport	13.26	5.75	10.95	5.39	12.49	5.46	2.72	0.068	0.03
	**Cluster 1**		**Cluster 2**		**Cluster 3**		**χ^2^ tests **		
	**n**	**%**	**n**	**%**	**n**	**%**	**df**	***χ^2^***	***p***
Sex							4	7.66	0.089 ^a^
Female	32	15.02	27	12.68	56	26.29			
Male	20	9.39	35	16.43	40	18.78			
Divers	2	0.94	0	0.00	1	0.47			
Sports type							2	1.99	0.371
Individual	28	13.15	25	11.74	40	18.78			
Team	26	12.21	37	17.37	57	26.76			
Participation in competitions							6	11.25	0.074 ^a^
Regularly	32	15.02	45	21.13	64	30.05			
Now and then	12	5.63	6	2.82	16	7.51			
Very rarely	4	1.88	2	0.94	12	5.63			
No competitions	6	2.82	9	4.23	5	2.35			
Performance level							6	1.76	0.966 ^a^
Professional level	0	0.00	1	0.47	1	0.47			
High performance level	6	2.82	4	1.88	8	3.76			
Competitive level	27	12.68	33	15.49	48	22.54			
Amateur level	21	9.86	24	11.27	40	18.78			
Competition level							4	3.96	0.402 ^a^
International	7	3.63	2	1.04	9	4.66			
National	11	5.70	14	7.25	19	9.84			
Regional	30	15.54	37	19.17	64	33.16			
Squad level							10	15.79	0.084 ^a^
Highest national level or Olympic squad	7	3.29	3	1.41	3	1.41			
Second highest national level or perspective or supplementary squad	4	1.88	0	0.00	9	4.23			
Third highest national level or junior squad	2	0.94	7	3.29	5	2.35			
Fourth highest national level or national squad	8	3.76	11	5.16	16	7.51			
Other lower competition level or other squad status	18	8.45	27	12.68	41	19.25			
No competition level or squad status	15	7.04	14	6.57	23	10.80			
National team							2	5.66	0.059
Yes	8	3.76	2	0.94	14	6.57			
No	46	21.60	60	28.17	83	38.97			
Mental training							2	3.15	0.207
Yes	7	3.29	6	2.82	19	8.92			
No	47	22.07	56	26.29	78	**36.62**			

Note. ^a^ Fisher’s exact test for count data.

**Table 8:** Means (M), standard deviations (SD), and 95% confidence intervals (95% CI) of psychosocial variables by cluster and the results of the respective ANOVAs.

	Cluster 1 (n = 54)			Cluster 2 (n = 62)			Cluster 3 (n = 97)			ANOVA between Group		
	M	SD	95% CI	M	SD	95% CI	M	SD	95% CI	*F*(2, 210)	*p*	η_p_²
**Personality**												
Athletic identity	5.38	0.91	[5.13; 5.63]	4.99	0.97	[4.74; 5.23]	5.19	1.06	[4.97; 5.40]	2.19	0.115	0.02
Excessive effort	3.34	0.61	[3.18; 3.51]	3.12	0.43	[3.01; 3.23]	3.16	0.47	[3.07; 3.26]	3.18	0.044	0.03
Internal control belief	4.09	0.68	[3.91; 4.28]	4.13	0.65	[3.96; 4.29]	4.29	0.65	[4.16; 4.42]	2.07	0.129	0.02
External control belief	2.40	0.78	[2.19; 2.61]	2.26	0.81	[2.05; 2.46]	2.29	0.62	[2.16; 2.41]	0.60	0.549	0.00
Resilience	3.21	0.78	[3.00; 3.43]	3.47	0.66	[3.30; 3.63]	3.43	0.66	[3.30; 3.57]	2.30	0.103	0.02
Sense of coherence ^a^	4.50	1.00	[4.18; 4.81]	4.92	0.94	[4.65; 5.18]	5.03	0.95	[4.82; 5.23]	4.27	0.015	0.05
**Competition anxiety**												
Somatic anxiety	2.76	0.77	[2.55; 2.97]	2.45	0.85	[2.24; 2.66]	2.61	0.83	[2.44; 2.78]	2.10	0.126	0.02
Concentration disruption	1.72	0.52	[1.58; 1.86]	1.78	0.63	[1.62; 1.94]	1.68	0.56	[1.57; 1.80]	0.57	0.564	0.00
Concerns	2.62	0.82	[2.40; 2.84]	2.52	0.71	[2.34; 2.70]	2.47	0.76	[2.32; 2.62]	0.69	0.504	0.00
Fear of (re-)injury	2.22	0.47	[2.09; 2.35]	2.17	0.37	[2.07; 2.26]	2.15	0.45	[2.06; 2.24]	0.50	0.605	0.00
**Stress(ors)**												
Life events	218.07	97.81	[191.38; 244.77]	217.65	91.52	[194.40; 240.89]	196.57	74.40	[181.57; 211.56]	1.62	0.200	0.02
Stress	2.35	0.50	[2.21; 2.48]	2.16	0.47	[2.04; 2.28]	2.11	0.46	[2.02; 2.20]	4.42	0.013	0.04
**Coping resources**												
Self-compassion	2.75	0.67	[2.56; 2.93]	3.18	0.64	[3.01; 3.34]	3.11	0.62	[2.99; 3.23]	7.85	<0.001	0.07
Mindfulness	3.76	0.75	[3.56; 3.97]	3.90	0.70	[3.73; 4.08]	4.06	0.63	[3.93; 4.19]	3.37	0.036	0.03
Social support	4.25	0.71	[4.05; 4.44]	4.34	0.66	[4.17; 4.51]	4.47	0.61	[4.35; 4.59]	2.15	0.119	0.02
Sport-specific self-efficacy	5.84	0.82	[5.61; 6.06]	5.42	0.81	[5.21; 5.62]	5.64	0.85	[5.47; 5.81]	3.74	0.025	0.03
Maladaptive coping	2.07	0.34	[1.98; 2.17]	2.07	0.39	[1.97; 2.16]	2.07	0.39	[2.00; 2.15]	0.93	0.992	0.00
Adaptive coping	2.52	0.45	[2.40; 2.64]	2.58	0.40	[2.48; 2.68]	2.65	0.41	[2.56; 2.73]	1.78	0.188	0.02

Note. Cluster 1 = currently and chronically injured athletes; Cluster 2 = athletes not seeking treatment; Cluster 3 = athletes utilizing medical treatment and rehabilitation; ^a^ n_Cluster 1_ = 41, n_Cluster 2_ = 50, n_Cluster 3_ = 82, n = 172.

## Data Availability

Data available in a publicly accessible repository: The data presented in this study are openly available in Open Science Framework at: osf.io/ctb9n (accessed on 17 November 2023).

## Supplementary Materials

The following supporting information can be downloaded at: https://www.mdpi.com/article/10.3390/sports11120237/s1. As supporting information, we created an overview about all measures assessed during the study. Supplement S1. Assessed demographics, sports- and injury-related information and used questionnaires.

## References

[1] Henke T., Luig P., Schulz D. (2014). Sportunfälle im Vereinssport in Deutschland: Aspekte der Epidemiologie und Prävention. Bundesgesundheitsblatt—Gesundheitsforschung—Gesundheitsschutz.

[2] Kisser R., Bauer R. (2012). The Burden of Sports Injuries in the European Union. Research Report D2h of the Project “Safety in Sports”.

[3] Ardern C.L., Glasgow P., Schneiders A., Witvrouw E., Clarsen B., Cools A., Gojanovic B., Griffin S., Khan K.M., Moksnes H. (2016). 2016 Consensus Statement on Return to Sport from the First World Congress in Sports Physical Therapy, Bern. Br. J. Sports Med..

[4] Bahr R., Clarsen B., Derman W., Dvorak J., Emery C.A., Finch C.F., Hägglund M., Junge A., Kemp S., International Olympic Committee Injury and Illness Epidemiology Consensus Group (2020). International Olympic Committee Consensus Statement: Methods for Recording and Reporting of Epidemiological Data on Injury and Illness in Sports 2020 (Including the STROBE Extension for Sports Injury and Illness Surveillance (STROBE-SIIS)). Orthop. J. Sports Med..

[5] Hreljac A. (2004). Impact and Overuse Injuries in Runners. Med. Sci. Sports Exerc..

[6] Putukian M. (2016). The Psychological Response to Injury in Student Athletes: A Narrative Review with a Focus on Mental Health. Br. J. Sports Med..

[7] Maffulli N., Longo U.G., Gougoulias N., Loppini M., Denaro V. (2010). Long-Term Health Outcomes of Youth Sports Injuries. Br. J. Sports Med..

[8] Wiese-Bjornstal D.M. (2010). Psychology and Socioculture Affect Injury Risk, Response, and Recovery in High-Intensity Athletes: A Consensus Statement: Sport Injury Psychology Consensus Statement. Scand. J. Med. Sci. Sports.

[9] Hägglund M., Waldén M., Magnusson H., Kristenson K., Bengtsson H., Ekstrand J. (2013). Injuries Affect Team Performance Negatively in Professional Football: An 11-Year Follow-up of the UEFA Champions League Injury Study. Br. J. Sports Med..

[10] Eliakim E., Morgulev E., Lidor R., Meckel Y. (2020). Estimation of Injury Costs: Financial Damage of English Premier League Teams’ Underachievement Due to Injuries. BMJ Open Sport Exerc. Med..

[11] Raysmith B.P., Drew M.K. (2016). Performance Success or Failure Is Influenced by Weeks Lost to Injury and Illness in Elite Australian Track and Field Athletes: A 5-Year Prospective Study. J. Sci. Med. Sport.

[12] Howden Group Holdings (2022). Howden’s European Football Injury Index Reveals Record Injury Cost of over £500m for 2021/22 Season. https://www.howdengroup.com/news-and-insights/howdens-european-footbal-Injury-Index-reveals-record-injury-cost-of-over-500m-for-2021-22-season.

[13] Ristolainen L., Kettunen J.A., Kujala U.M., Heinonen A. (2012). Sport Injuries as the Main Cause of Sport Career Termination among Finnish Top-Level Athletes. Eur. J. Sport Sci..

[14] te Wierike S.C.M., van der Sluis A., van den Akker-Scheek I., Elferink-Gemser M.T., Visscher C. (2013). Psychosocial Factors Influencing the Recovery of Athletes with Anterior Cruciate Ligament Injury: A Systematic Review: Psychosocial Influences on Recovery of ACL Injury. Scand. J. Med. Sci. Sports.

[15] Ivarsson A., Johnson U., Andersen M.B., Tranaeus U., Stenling A., Lindwall M. (2017). Psychosocial Factors and Sport Injuries: Meta-Analyses for Prediction and Prevention. Sports Med.

[16] Li S., Wu Q., Chen Z. (2020). Effects of Psychological Interventions on the Prevention of Sports Injuries: A Meta-Analysis. Orthop. J. Sports Med..

[17] Tranaeus U., Ivarsson A., Johnson U. (2015). Evaluation of the Effects of Psychological Prevention Interventions on Sport Injuries: A Meta-Analysis. Sci. Sports.

[18] Bahr R., Clarsen B., Ekstrand J. (2018). Why We Should Focus on the Burden of Injuries and Illnesses, Not Just Their Incidence. Br. J. Sports Med..

[19] Yang J., Tibbetts A.S., Covassin T., Cheng G., Nayar S., Heiden E. (2012). Epidemiology of Overuse and Acute Injuries Among Competitive Collegiate Athletes. J. Athl. Train..

[20] Forsdyke D., Smith A., Jones M., Gledhill A. (2016). Psychosocial Factors Associated with Outcomes of Sports Injury Rehabilitation in Competitive Athletes: A Mixed Studies Systematic Review. Br. J. Sports Med..

[21] Williams J.M., Andersen M.B. (1998). Psychosocial Antecedents of Sport Injury: Review and Critique of the Stress and Injury Model’. J. Appl. Sport Psychol..

[22] Clement D., Ivarsson A., Tranaeus U., Johnson U., Stenling A. (2018). Investigating the Influence of Intraindividual Changes in Perceived Stress Symptoms on Injury Risk in Soccer. Scand. J. Med. Sci. Sports.

[23] Ivarsson A., Johnson U., Podlog L. (2013). Psychological Predictors of Injury Occurrence: A Prospective Investigation of Professional Swedish Soccer Players. J. Sport Rehabil..

[24] Rogers T.J., Landers D.M. (2005). Mediating Effects of Peripheral Vision in the Life Event Stress/Athletic Injury Relationship. J. Sport Exerc. Psychol..

[25] Johnson U., Ivarsson A. (2013). Stressors as Antecedents to Sports Injuries: A Psychological Perspective. Advances in Psychology Research, Volume 97.

[26] Steffen K., Pensgaard A.M., Bahr R. (2009). Self-Reported Psychological Characteristics as Risk Factors for Injuries in Female Youth Football. Scand. J. Med. Sci. Sports.

[27] Brewer B.W., Van Raalte J.L., Linder D.E. (1993). Athletic Identity: Hercules’ Muscles or Achilles Heel?. Int. J. Sport Psychol..

[28] Schuster M. (2011). Einfluss des Motivationalen Trainingsklimas auf die Exzessive Verausgabungsbereitschaft und dem Damit Verbundenen Verletzungsrisiko. Master’s Thesis.

[29] Kovaleva A. (2012). The IE-4: Construction and Validation of a Short Scale for the Assessment of Locus of Control.

[30] Chmitorz A., Wenzel M., Stieglitz R.-D., Kunzler A., Bagusat C., Helmreich I., Gerlicher A., Kampa M., Kubiak T., Kalisch R. (2018). Population-Based Validation of a German Version of the Brief Resilience Scale. PLoS ONE.

[31] Kennedy P., Lude P., Elfström M.L., Smithson E. (2010). Sense of Coherence and Psychological Outcomes in People with Spinal Cord Injury: Appraisals and Behavioural Responses. Br. J. Health Psychol..

[32] Martens R., Vealey R.S., Burton D. (1990). Competitive Anxiety in Sport.

[33] Kori S., Miller R., Todd D. (1990). Kinesiophobia: A New View of Chronic Pain Behavior. Pain Manag..

[34] Holmes T.H., Rahe R.H. (1967). The Social Readjustment Rating Scale. J. Psychosom. Res..

[35] Fliege H., Rose M., Arck P., Walter O.B., Kocalevent R.-D., Weber C., Klapp B.F. (2005). The Perceived Stress Questionnaire (PSQ) Reconsidered: Validation and Reference Values From Different Clinical and Healthy Adult Samples. Psychosom. Med..

[36] Hupfeld J., Ruffieux N. (2011). Validierung einer deutschen Version der Self-Compassion Scale (SCS-D). Z. Für Klin. Psychol. Und Psychother..

[37] Höfling V., Moosbrugger H., Schermelleh-Engel K., Heidenreich T. (2011). Mindfulness or Mindlessness?: A Modified Version of the Mindful Attention and Awareness Scale (MAAS). Eur. J. Psychol. Assess..

[38] Sarason I.G., Levine H.M., Basham R.B., Sarason B.R. (1983). Assessing Social Support: The Social Support Questionnaire. J. Personal. Soc. Psychol..

[39] Fuchs R., Schwarzer R. (1994). Selbstwirksamkeit Zur Sportlichen Aktivität: Reliabilität Und Validität Eines Neuen Messinstruments. [Self-Efficacy toward Physical Exercise: Reliability and Validity of a New Instrument.. Z. Für Differ. Und Diagn. Psychol..

[40] Knoll N., Rieckmann N., Schwarzer R. (2005). Coping as a Mediator between Personality and Stress Outcomes: A Longitudinal Study with Cataract Surgery Patients. Eur. J. Personal..

[41] Smith R.E., Smoll F.L. (1995). Development and Validation of a Multidimensional Measure of Sport-Specific Psychological Skills: The Athletic Coping Skills Inventory-28. J. Sports Exerc. Psychol..

[42] Devantier C. (2011). Psychological Predictors of Injury among Professional Soccer Players. Sport Sci. Rev..

[43] Fernandes H.M., Reis V.M., Vilaça-Alves J., Saavedra F., Aidar F.J., Brustad R. (2014). Social Support and Sport Injury Recovery: An Overview of Empirical Findings and Practical Implications. Rev. Psicol. Deporte.

[44] Forsdyke D., Madigan D., Gledhill A., Smith A. (2022). Perceived Social Support, Reinjury Anxiety, and Psychological Readiness to Return to Sport in Soccer Players. J. Sport Rehabil..

[45] Hardy C.J., Richman J.M., Rosenfeld L.B. (1991). The Role of Social Support in the Life Stress/Injury Relationship. Sport Psychol..

[46] Smith R.E., Smoll F.L., Ptacek J.T. (1990). Conjunctive Moderator Variables in Vulnerability and Resiliency Research: Life Stress, Social Support and Coping Skills, and Adolescent Sport Injuries. J. Personal. Soc. Psychol..

[47] Bartlett L., Martin A., Neil A.L., Memish K., Otahal P., Kilpatrick M., Sanderson K. (2019). A Systematic Review and Meta-Analysis of Workplace Mindfulness Training Randomized Controlled Trials. J. Occup. Health Psychol..

[48] Ferrari M., Hunt C., Harrysunker A., Abbott M.J., Beath A.P., Einstein D.A. (2019). Self-Compassion Interventions and Psychosocial Outcomes: A Meta-Analysis of RCTs. Mindfulness.

[49] Jayawardene W.P., Lohrmann D.K., Erbe R.G., Torabi M.R. (2017). Effects of Preventive Online Mindfulness Interventions on Stress and Mindfulness: A Meta-Analysis of Randomized Controlled Trials. Prev. Med. Rep..

[50] Phillips W.J., Hine D.W. (2021). Self-Compassion, Physical Health, and Health Behaviour: A Meta-Analysis. Health Psychol. Rev..

[51] Ivarsson A., Johnson U., Andersen M.B., Fallby J., Altemyr M. (2015). It Pays to Pay Attention: A Mindfulness-Based Program for Injury Prevention With Soccer Players. J. Appl. Sport Psychol..

[52] Naderi A., Shaabani F., Gharayagh Zandi H., Calmeiro L., Brewer B.W. (2020). The Effects of a Mindfulness-Based Program on the Incidence of Injuries in Young Male Soccer Players. J. Sport Exerc. Psychol..

[53] Huysmans Z., Clement D. (2017). A Preliminary Exploration of the Application of Self-Compassion Within the Context of Sport Injury. J. Sport Exerc. Psychol..

[54] Johnson K.L., Cormier D.L., Kowalski K.C., Mosewich A.D. (2022). Exploring the Relationship Between Mental Toughness and Self-Compassion in the Context of Sport Injury. J. Sport Rehabil..

[55] Wesch N., Hall C., Prapavessis H., Maddison R., Bassett S., Foley L., Brooks S., Forwell L. (2012). Self-Efficacy, Imagery Use, and Adherence during Injury Rehabilitation: Self Efficacy & Imagery Use in Rehabilitation. Scand. J. Med. Sci. Sports.

[56] Cagle J.A., Overcash K.B., Rowe D.P., Needle A.R. (2017). Trait Anxiety as a Risk Factor for Musculoskeletal Injury in Athletes: A Critically Appraised Topic. Int. J. Athl. Ther. Train..

[57] Ford J., Ildefonso K., Jones M., Arvinen-Barrow M. (2017). Sport-Related Anxiety: Current Insights. Open Access J. Sports Med..

[58] Li H., Moreland J.J., Peek-Asa C., Yang J. (2017). Preseason Anxiety and Depressive Symptoms and Prospective Injury Risk in Collegiate Athletes. Am. J. Sports Med..

[59] Kolt G., Kirkby R. (1996). Injury in Australian Female Competitive Gymnasts: A Psychological Perspective. Aust. J. Physiother..

[60] Pargman D., Lunt S.D. (1989). The Relationship of Self-concept and Locus of Control to the Severity of Injury in Freshmen Collegiate Football Players. Sports Med. Train. Rehabil..

[61] Kerr G., Minden H. (1988). Psychological Factors Related to the Occurrence of Athletic Injuries. J. Sport Exerc. Psychol..

[62] Codonhato R., Rubio V., Oliveira P.M.P., Resende C.F., Rosa B.A.M., Pujals C., Fiorese L. (2018). Resilience, Stress and Injuries in the Context of the Brazilian Elite Rhythmic Gymnastics. PLoS ONE.

[63] Wadey R., Evans L., Hanton S., Neil R. (2012). An Examination of Hardiness throughout the Sport Injury Process: Sport Injury Process. Br. J. Health Psychol..

[64] Mayer J., Thiel A. (2014). Health in Elite Sports from a Salutogenetic Perspective: Athletes’ Sense of Coherence. PLoS ONE.

[65] Renton T., Petersen B., Kennedy S. (2021). Investigating Correlates of Athletic Identity and Sport-Related Injury Outcomes: A Scoping Review. BMJ Open.

[66] Würth S., Henke T., Schulz D., Platen P. (2014). Verausgabungsbereitschaft Als Prädiktor Für Verletzungen Im Handball. Sicherheit im Sport–Ein Leben mit Sport–aber Sicher.

[67] Würth S. (2014). Verausgabungsbereitschaft Und Overconformity Im Kontext von Verletzungen Im Sport.

[68] Leiner D.J. (2019). SoSci Survey.

[69] World Medical Association (2013). Declaration of Helsinki-Ethical Principles for Medical Research Involving Human Subjects. JAMA.

[70] Dalmaijer E.S., Nord C.L., Astle D.E. (2022). Statistical Power for Cluster Analysis. BMC Bioinform..

[71] Faul F., Erdfelder E., Lang A.-G., Buchner A. (2007). G*Power 3: A Flexible Statistical Power Analysis Program for the Social, Behavioral, and Biomedical Sciences. Behav. Res. Methods.

[72] Schmid J., Seiler R. (2003). Identität im Hochleistungssport. Diagnostica.

[73] Würth S., Amesberger G., Theodorakis Y., Papaioannou A., Goudas M. (2007). Excessive Effort in Sport–Development and Validation of the Excessive Effort in Sport Scale (EESS). European Federation of Sport Psychology, 12th European Congress of Sport Psychologe. Sport and Exercise Psychology: Bridges between Disciplines and Cultures. Long Papers.

[74] Schumacher J., Wilz G., Gunzelmann T., Brähler E. (2000). Die Sense of Coherence Scale von Antonovsky—Teststatistische Überprüfung in einer repräsenta-tiven Bevölkerungsstichprobe und Konstruktion einer Kurzskala-. Psychother. Psychosom. Med. Psychol..

[75] Brand R., Ehrenspiel F., Graf K. (2009). Wettkampf-Angst-Inventar (WAI) Manual Zur Komprehensiven Eingangsdiagnostik von Wettkampfangst, Wettkampfängstlichkeit Und Angstbewältigungsmodus Im Sport.

[76] Rusu A.C., Kreddig N., Hallner D., Hülsebusch J., Hasenbring M.I. (2014). Fear of Movement/(Re)Injury in Low Back Pain: Confirmatory Validation of a German Version of the Tampa Scale for Kinesiophobia. BMC Musculoskelet. Disord..

[77] Raes F., Pommier E., Neff K.D., Van Gucht D. (2011). Construction and Factorial Validation of a Short Form of the Self-Compassion Scale. Clin. Psychol. Psychother..

[78] Fydrich T., Sommer G., Tydecks S., Brähler E. (2009). Fragebogen zur sozialen Unterstützung (F-SozU): Normierung der Kurzform (K-14). Z. Für Med. Psychol..

[79] (2022). R Core Team R: A Language and Environment for Statistical Computing.

[80] (2023). Posit Team RStudio: Integrated Development Environment for R.

[81] Kaufman L., Rousseeuw P.J. (1990). Finding Groups in Data: An Introduction to Cluster Analysis.

[82] Gower J.C. (1971). A General Coefficient of Similarity and Some of Its Properties. Biometrics.

[83] Rousseeuw P.J. (1987). Silhouettes: A Graphical Aid to the Interpretation and Validation of Cluster Analysis. J. Comput. Appl. Math..

[84] Field A.P., Miles J., Field Z. (2012). Discovering Statistics Using R.

[85] Cohen J. (1988). Statistical Power Analysis for the Behavioral Sciences.

[86] Blanca M.J., Alarcón R., Arnau J. (2017). Non-Normal Data: Is ANOVA Still a Valid Option?. Psicothema.

[87] Finch H. (2005). Comparison of the Performance of Nonparametric and Parametric MANOVA Test Statistics When Assumptions Are Violated. Methodology.

[88] Holm S. (1979). A Simple Sequentially Rejective Multiple Test Procedure. Scand. J. Stat..

[89] Ivarsson A., Johnson U., Lindwall M., Gustafsson H., Altemyr M. (2014). Psychosocial Stress as a Predictor of Injury in Elite Junior Soccer: A Latent Growth Curve Analysis. J. Sci. Med. Sport.

[90] Shuer M.L., Dietrich M.S. (1997). Psychological Effects of Chronic Injury in Elite Athletes. West. J. Med..

[91] Hamilton L.H., Hamilton W.G., Meltzer J.D., Marshall P., Molnar M. (1989). Personality, Stress, and Injuries in Professional Ballet Dancers. Am. J. Sports Med..

[92] Llewellyn D.J., Sanchez X. (2008). Individual Differences and Risk Taking in Rock Climbing. Psychol. Sport Exerc..

[93] Castro Sánchez M., Chacón Cuberos R., Zurita Ortega F., Espejo Garcés T. (2015). Niveles de resiliencia en base a modalidad, nivel y lesiones deportivas (Levels of resilience based on sport discipline, competitive level and sport injuries). Retos.

[94] Gledhill A., Forsdyke D. (2021). The Psychology of Sport Injury: From Risk to Retirement.

[95] Kolar E., Pavletič M.S., Smrdu M., Atiković A. (2017). Athletes’ Perception of the Causes of Injury in Gymnastics. J. Sports Med. Phys. Fit..

[96] Johnson U., Tranaeus U., Ivarsson A. (2014). Current Status and Future Challenges in Psychological Research of Sport Injury Prediction and Prevention: A Methodological Perspective. Rev. Psicol. Deporte.

[97] Bittencourt N.F.N., Meeuwisse W.H., Mendonça L.D., Nettel-Aguirre A., Ocarino J.M., Fonseca S.T. (2016). Complex Systems Approach for Sports Injuries: Moving from Risk Factor Identification to Injury Pattern Recognition—Narrative Review and New Concept. Br. J. Sports Med..

[98] Pensgaard A.M., Ivarsson A., Nilstad A., Solstad B.E., Steffen K. (2018). Psychosocial Stress Factors, Including the Relationship with the Coach, and Their Influence on Acute and Overuse Injury Risk in Elite Female Football Players. BMJ Open Sport Exerc. Med..

[99] Tranaeus U., Johnson U., Engström B., Skillgate E., Werner S. (2014). Psychological Antecedents of Overuse Injuries in Swedish Elite Floorball Players. Athl. Insight.

[100] Hill Y., Den Hartigh R.J.R. (2023). Resilience in Sports through the Lens of Dynamic Network Structures. Front. Netw. Physiol..

