# Supplementary material for: Not All Injuries Are the Same: Different Patterns in Sports Injuries and Their Psychosocial Correlates
Source: Sports (Basel). 2023 Dec 1;11(12):237. doi: 10.3390/sports11120237 (PMC10747018; doi:10.3390/sports11120237)
Supplement: Supplementary file 1 [file sports-11-00237-s001.zip › sports-2663098-Supplementary Table S1.pdf]

Supplement Table S1. Assessed demographics, sports- and injury-related information and used questionnaires.

**Demographic data**	• Sex<br>• Age<br>• Graduation level<br>• Occupation<br>• Study course<br>• Semesters of study
**Sports-related data**	• Sport<br>• Type of sports<br>• Training sessions in main sport<br>• Additional training sessions<br>• Hours of training per week<br>• Competition participation<br>• Competition level<br>• Performance level<br>• Squad level<br>• Number of years conducting the sport<br>• Seasonal phase<br>• Part of national team<br>• Experience with mental training
**Injury-related data**	• Current injury status<br>• *Description of injury*<br>• Frequency within the last 12 months<br>• *Setting of current or past injury*<br>• *Date of current or past injury*<br>• *Cause of current or past injury*<br>• Severity of current or past injury<br>• Medical treatment of current or past injury<br>• *Drug treatment of current or past injury*<br>• Rehabilitation measures of current or past injury<br>• *Achievement of pre-injury performance level (only past injuries)*<br>• *Recovery time (only past injuries)*<br>• *Consequences of current or past injury*<br>• Chronicity
**Psychosocial data**	• Life Events (SRRS)<br>• Perceived Stress (PSQ)<br>• *Depression and Anxiety (STADI)*<br>• *Worry (PSWQ)*<br>• *Mental Health (GHQ)*<br>• *Fatigue (FAS)*<br>• Competition anxiety (WAI-T)<br>• Locus of Control (IE-4)<br>• *Rumination (PTQ)*<br>• Mindfulness (MAAS)<br>• Social Support (FSoz-KU14)<br>• *Burnout (ABQ-D)*<br>• *Sports-related rumination (SCRS)*<br>• Self-Compassion (SCS-D short scale)<br>• Fear of (re-)injury (TSK)<br>• Athletic Identity (AIMS-D)<br>• Willingness to spend (EESS)<br>• Self-Efficacy (ESES)<br>• Coping behavior (Brief COPE)<br>• Resilience (BRS)<br>• Sense of Coherence (SOC-L9)

Note. Information and questionnaires **not** used in the data analyses of the present study are italic.
